# The Effects of Ecstasy (MDMA) on Brain Serotonin Transporters Are Dependent on Age-of-First Exposure in Recreational Users and Animals

**DOI:** 10.1371/journal.pone.0047524

**Published:** 2012-10-24

**Authors:** Anne Klomp, Bjørnar den Hollander, Kora de Bruin, Jan Booij, Liesbeth Reneman

**Affiliations:** 1 Brain Imaging Center, Academic Medical Center, Amsterdam, The Netherlands; 2 Department of Radiology, Academic Medical Center, Amsterdam, The Netherlands; 3 Department of Nuclear Medicine, Academic Medical Center, Amsterdam, The Netherlands; University G. D’Annunzio, Italy

## Abstract

**Rationale and Objective:**

Little is known on the effects of ecstasy (MDMA, a potent 5-HT-releaser and neurotoxin) exposure on brain development in teenagers. The objective of this study was to investigate whether in humans, like previous observations made in animals, the effects of MDMA on the 5-HT system are dependent on age-of-first exposure.

**Methods:**

5-HT transporter (SERT) densities in the frontal cortex and midbrain were assessed with [^123^I]β-CIT single photon emission computed tomography in 33 users of ecstasy. Subjects were stratified for early-exposed users (age-at-first exposure 14–18 years; developing brain), and late-exposed users (age-at-first exposure 18–36 years; mature brain). In parallel, we investigated the effects of age experimentally with MDMA in early-exposed (adolescent) rats and late-exposed (adult) rats using the same radioligand.

**Results:**

On average, five years after first exposure, we found a strong inverse relationship, wherein age-at-first exposure predicted 79% of the midbrain SERT variability in early (developing brain) exposed ecstasy users, whereas this was only 0.3% in late (mature brain) exposed users (p = 0.007). No such effect was observed in the frontal cortex. In rats, a significant age-BY-treatment effect (p<0.01) was observed as well, however only in the frontal cortex.

**Conclusions:**

These age-related effects most likely reflect differences in the maturational stage of the 5-HT projection fields at age-at-first exposure and enhanced outgrowth of the 5-HT system due to 5-HT’s neurotrophic effects. Ultimately, our findings stress the need for more knowledge on the effects of pharmacotherapies that alter brain 5-HT levels in the pediatric population.

## Introduction

The monoamine neurotransmitter serotonin (5-HT) plays a key role in the development of the central nervous system through its role in the connective organization of the brain. It is known not only to autoregulate the outgrowth of serotonergic neurons but has also been implicated in the control of cell proliferation, differentiation, migration, cell death, synaptogenesis and dendritic pruning [1]–[3]. The brain in development is dependent on the emergence of these critical developmental processes and is thus sensitive to pharmacological interventions that can influence those. In this way, substances that induce heightened levels of 5-HT can lead to disturbed outgrowth of the 5-HT system when administered during (early) brain development [4]–[7]. This also includes the drug of abuse 3,4-methylenedioxymethamphetamine (MDMA, ecstasy), a strong 5-HT releasing agent which can lead to enhanced 5-HT outgrowth in case of fetal exposure [8], [9]. This is striking, since dose-dependent reductions in 5-HT markers such as the 5-HT transporter (SERT) are observed in adult animals [10], and probably also in humans [11]. Studies examining the effects of perinatal MDMA exposure also show that immature animals are less susceptible than adults to the neurotoxic effects of MDMA [12]–[14]. These neurotoxic effects encompass long-term and long-lasting reductions in several markers of the 5-HT system (for example lower 5-HT and its metabolite 5-hydroxyindoleacetic acid (5-HIAA) concentrations, SERT density, less activity of the rate-limiting 5-HT synthesis enzyme tryptophan hydroxylase (TPH) and loss of 5-HT axons) in animals [15], [16]. As said before, MDMA seems to have much less of these neurotoxic effects on the perinatal brain. In rats, prenatal exposure has been shown not to affect any 5-HT markers [14], [17], while postnatally 5-HT sensitivity to MDMA seems to develop only after PND35 [12], [14]. Even during adolescent exposure, MDMA has been shown to reduce 5-HT transporter (SERT) densities in the frontal cortex still ‘only’ by 21%, which is less pronounced than the 62% reduction seen in adult animals [12]. Although human studies are less abundant, there is also evidence of reduced levels of 5-HIAA and SERT densities in ecstasy abusers. 5-HT2A receptor availability has found to be lower after recent abuse, but higher in former ecstasy abusers, perhaps due to compensatory receptor synthesis in response to 5-HT depletion [11]. Although MDMA is known to directly affect the dopamine (DA) and noradrenaline (NA) systems as well, albeit to a lesser extend than 5-HT, no long-term neurotoxic effects on other monoamine neurotransmitter systems have been found so far [15], [16].

As mentioned before, the developing brain is sensitive to pharmacological interventions that influence normal neurotransmitter function. The perinatal period is not the only critical period though; the periadolescent period is characterized by a remarkable overshoot of synapses and neurotransmitter receptors, followed by synaptic pruning and maturation of remaining connections [18]. The monoamine neurotransmitters, such as 5-HT, play an important role in these processes as well [19]. Animal studies have shown that 5-HT neurotransmission undergoes widespread remodeling from early youth through adolescence into adulthood [20], [21]. During this entire period the number of 5-HT synapses is known to fluctuate, a steady increase of SERT is seen, mainly in the frontal cortex, and also a clear reorganization of 5-HT receptor expression [20], [22]. Hence, the brain is in another critical period of development during adolescence and is therefore thought to be more vulnerable to drug exposure in this period [23], while it is this period in which many youngsters experiment with drugs of abuse, such as MDMA, due to increased risk-taking behaviour and reduced impulsive control associated with these pubertal changes [24].

However, little is still known on the effects of altered 5-HT expression on (late) brain development, especially in humans. One way to study the effects of 5-HT alterations on neuronal development in humans is by examining the 5-HT system in recreational users of the ecstasy who have been exposed early during adolescence (developing brain), compared to users only exposed during adulthood (mature brain), with ecstasy use of course being a rather extreme example of 5-HT manipulation. There are some indications from the literature that adolescent MDMA exposure exerts different effects than adult exposure. For example, 2-[18F]fluoro-2-deoxy-D-glucose (FDG) PET imaging studies have reported altered glucose metabolism in MDMA users, which seems to be more severe in subjects exposed to MDMA before the age of 18 [15].

As SERT is a structural component of the 5-HT system, and has been validated as a reliable marker for the integrity of the 5-HT system, we aimed at measuring SERT binding in recreational users of the party drug ecstasy in which one group has been exposed during adolescence (developing brain), and the other group during adulthood (mature brain). In parallel, we investigated the effects of MDMA on SERT density in early-exposed (adolescent) rats and late-exposed (adult) rats. *In rats*, under normal rearing conditions, SERT densities in the midbrain already reach a plateau at weaning. In ontogenetically later maturing brain regions, such as the frontal cortex, SERT increases steadily from weaning till old age [22], [25], [26]. *In humans*, on the other hand, midbrain SERT decreases with increasing age after the age of 18, as assessed with single photon emission computed tomography (SPECT) [27], whereas before the age of 18, no age-associated changes have been observed [28]. With respect to the frontal cortex, most studies showed no aging effect of SERT [29], [30], although the dorsal aspects of the frontal lobe are known to be the latest to mature [31].

The objective of this study was to investigate whether in humans and in rats the effects of MDMA on the 5-HT system are dependent on age-of-first exposure, like previous observations made in animals [12]. Based on the essential role of SERT in the integrity of the 5-HT system and on the literature on normal age-associated changes in SERT density in rodents and humans, we hypothesized the following: a) *in the midbrain*, ecstasy induced reductions in SERT binding will be less pronounced in subjects exposed to ecstasy relatively late (matured brain) when compared to adolescence (maturing brain), due to a decrease in SERT with increasing age after the age of 18; b) No age-related effects of MDMA in rats, due to early maturation of this brain region in rat; c) *in the frontal cortex*, on the other hand, we expect no age-related effects of ecstasy exposure in the human subjects; d) but in rats we expect less pronounced effects following early exposure when compared to adult exposure, due to an increase in SERT with increasing age.

## Methods and Materials

### Human Subjects

The present study population was described in previous publications in which we investigated the effects of ecstasy on SERT [11], [32]. Exclusion criteria were: positive drug screen; pregnancy; severe medical or neuropsychiatric illness that precluded informed consent; and lifetime psychiatric disorder. Subjects were interviewed with the computer assisted version of the Composite International Diagnostic Interview (CIDI, v2.1.) to screen for the presence of DSM-IV mental disorders. Subjects selected for this study were between 18 and 45 years old at the time of study entrance, otherwise healthy, and with no psychiatric history. The ecstasy users were stratified based upon age of first ecstasy use: one group consisted of 8 subjects who had started using ecstasy before the age of 18 (‘early exposure’ group), and the other group consisted of 25 subjects that were older than 18 years of age when starting ecstasy use (‘late exposure’ group). The cut off point of 18 years of age was chosen based on the literature showing age-associated changes in midbrain SERT in subjects aged 18 years and older [27], [28]. In humans, on the other hand, SERT decreases with increasing age after the age of 18. All participants agreed to abstain from use of psychoactive drugs (including MDMA) for at least 3 weeks prior to the study, and were asked to undergo urine drug screening to assess current exposure to psychoactive drugs before enrolment. Subjects were informed that reimbursement for participation was contingent on no evidence of drug use on the urine sample. The institutional Medical Ethics Committee of the Academic Medical Center in Amsterdam approved the study. After complete description of the study to the subjects, written informed consent was obtained from all participants. This study was conducted according to the principles expressed in the Declaration of Helsinki.

### SERT Imaging Studies

The subjects in each group were examined using SPECT with the SERT ligand ^123^I-labelled 2β-carbomethoxy-3β-(4-iodophenyl) tropane ([^123^I]β-CIT). β-CIT binds with high affinity to both dopamine and 5-HT transporters, and has made it possible to assess the density of cortical and subcortical SERT in the living human brain, using SPECT [33]. β-CIT has been shown to adequately detect changes in midbrain SERT and frontal cortex SERT secondary to MDMA treatment in non-human primate using SPECT [34]. [^123^I]β-CIT binding in the cerebral cortex is predominantly to SERT, as pretreatment with the SSRI paroxetine significantly reduces [^123^I]β-CIT binding in the prefrontal cortex in rats and humans [35], [36].

SPECT studies were acquired in all subjects with a brain dedicated SPECT system (Strichman Medical Equipment 810X, Strichman Medical Equipment Inc., Medfield, Mass., USA). This 12-detector single-slice scanner has a full width at half maximum (FWHM) resolution of approximately 7.5 mm. Transversal slices parallel to and upward in 5 mm steps from the orbito-meatal (OM) line to the vertex were acquired after positioning of the subject in the camera with a fixed light source oriented along the OM line. Each acquisition consisted of approximately 15 slices, (acquired in a 64×64 matrix) with 3 minutes scanning time per slice. The energy window was set at 135–190 keV. Acquisition was commenced 4 h after iv injection of approximately 140 MBq [^123^I]β-CIT, a time when specific binding to SERT is at a maximum and stable for up to 10 h after injection [37]. Reconstruction and attenuation correction of all images were performed as previously described [36].

An investigator unaware of the participant’s history carried out a region-of-interest analysis using a standard template for the frontal cortex, midbrain and cerebellum constructed manually from co-registered MR images in 4 control subjects. The binding in the cerebellum, which is presumed to be low in SERT, was used as a reference for background radioactivity (non-specific binding + free ligand). The ratios of specific to non-specific [^123^I]β-CIT binding in midbrain and the frontal cortex were calculated by dividing specific frontal cortex and midbrain binding by unspecific binding in the cerebellum, which is a well-established measure for assessment of SERT density [38], [39].

### Animals and Drug Treatment

Male Wistar rats were obtained from Harlan (Horst, Netherlands). Animals were housed in a temperature and humidity controlled environment with food and water available ad libitum. The age at the start of the experimental manipulations in adolescent rats was PND27 (+/−0 days). This specific age was selected because adolescence is thought to last from PND28 to PND60 in male rats [24]. Treatment in adult animals started at PND63 (+/−5 days). Groups of rats (n = 5–8) were given either a vehicle or a neurotoxic regimen of MDMA which consisted of a subcutaneous dose of 10 mg/kg MDMA given twice daily for four consecutive days, followed by a seven day washout period. MDMA (certified reference compound, purity 98.9%) was obtained from the Netherlands Forensic Institute (Rijswijk, the Netherlands). All experiments involving procedures using animals were approved by the local Animal Care Committee at the Academic Medical Center in Amsterdam, according to relevant national and international guidelines.

### SERT Binding Studies

MDMA and vehicle-treated rats were injected intravenously with [^123^I]ß-CIT seven days after the last treatment (PND38 in adolescent animals and PND74 (+/−5 days) in adult animals). Three hours after injection of [^123^I]ß-CIT [40], animals were killed by bleeding via heart puncture under carbon dioxide anesthesia. The brains were quickly removed and dissected into the following regions: prefrontal cortex, temporal cortex, occipital cortex, hypothalamus, amygdala, hippocampus, striatum and cerebellum and weighed. For the midbrain, the superior colliculus was dissected because [^123^I]β-CIT uptake in the brainstem is highest in the superior colliculus in non-human primates [27]. The striatum was included as a negative control, since previous studies have failed to find an effect of MDMA using [^123^I]ß-CIT in this brain region, since it mainly reflects binding to dopamine transporters. Radioactivity of [^123^I]ß-CIT in each region was assayed with a gamma counter. The data were corrected for radioactivity decay back to the time of preparation of the injection syringes in order to compare relative concentrations in the tissues taken and to relate the results to the injected dose. The amount of radioactivity was expressed as a percentage of the injected dose, multiplied by the body weight per gram tissue weight (% ID×kg/g tissue), as described previously [41]. Binding of [^123^I]ß-CIT to SERT was analyzed using the ratio of specific to non-specific binding, using the cerebellum as a reference region for background radioactivity [39].

### Statistics

Data were tested for normality using the Kolmogorov-Smirnov test. In the human experiment, differences in continuous variables (log-transformed if necessary) between the two groups were analyzed using a Student *t*-test. The association between midbrain and frontal cortex SERT and age-at-first ecstasy exposure was assessed using Spearman’s correlation coefficient, regression analysis and univariate analysis of variance (ANOVA). Possible confounding variables such as gender, total amount of ecstasy intake, duration of intake, time since last exposure, and exposure to other drugs of abuse on SERT expression were assessed with univariate ANOVAs as well.

In the experiments in rodents, group differences in SERT densities were analyzed using multivariate ANOVA, with SERT density in all assessed brain regions as dependent variables and age and treatment as independent variables, followed by Bonferroni post-hoc tests when appropriate. Main age, treatment and age-BY-treatment interactions effects were further analyzed using a Student *t*-test. The chance of a type I error (α) was set at 0.05 using 2-tailed tests of significance. All data were analyzed using SPSS version 16.0.

## Results

### Demographics

Mean age-at-first ecstasy use was 16.1 years in the early-exposed group versus 22.4 years in the late-exposed group (p<0.01). On average subjects were scanned 4.7 years after their first ecstasy tablet in the early-exposed group and 5.2 years in the late-exposed group (p = NS). Apart from age at scanning (20.5 years in the early-exposed group versus 27.5 years in the late-exposed group, p<0.01) no differences were noted between the two groups: gender distribution, total ecstasy exposure, time since last exposure, as well as exposure to other drugs (alcohol, tobacco, cannabis, amphetamine and cocaine) was very similar for the two groups. An overview of these demographics is given in Table 1.

**Table 1:** Demographics, characteristics of MDMA use and exposure to other recreational drugs.

	First ecstasy <18 years	First ecstasy ≥18 years	
	n = 8	n = 25	p-values
Age	20.5 (1.7)	27.5 (5.7)	<0.01
Gender (M/F)	4/4	13/12	NS
*Ecstasy use*			
Lifetime no. tablets	148 (190)	272 (476)	NS
Time since last dose (months)	1.4 (0.8)	3.5 (4.9)	NS
Age first use (years)	16.1 (1.0)	22.4 (4.7)	<0.01
Duration of use (years)	4.7 (1.4)	5.2 (3.1)	NS
Usual dose (mg)	193.8 (72.9)	180 (79.1)	NS
*Last 3 months use of*			
Alcohol (units/week)	10.1 (10.9)	11.2 (9.9)	NS
Tobacco (cig./day)	9.4 (7.8)	9.2 (9.9)	NS
Cannabis (no. joints)	39.5 (52.4)	68.1 (94.4)	NS
Amphetamine (g)	3.5 (5.3)	2.2 (5.8)	NS

### SERT Imaging in Humans

**Figure 1:**
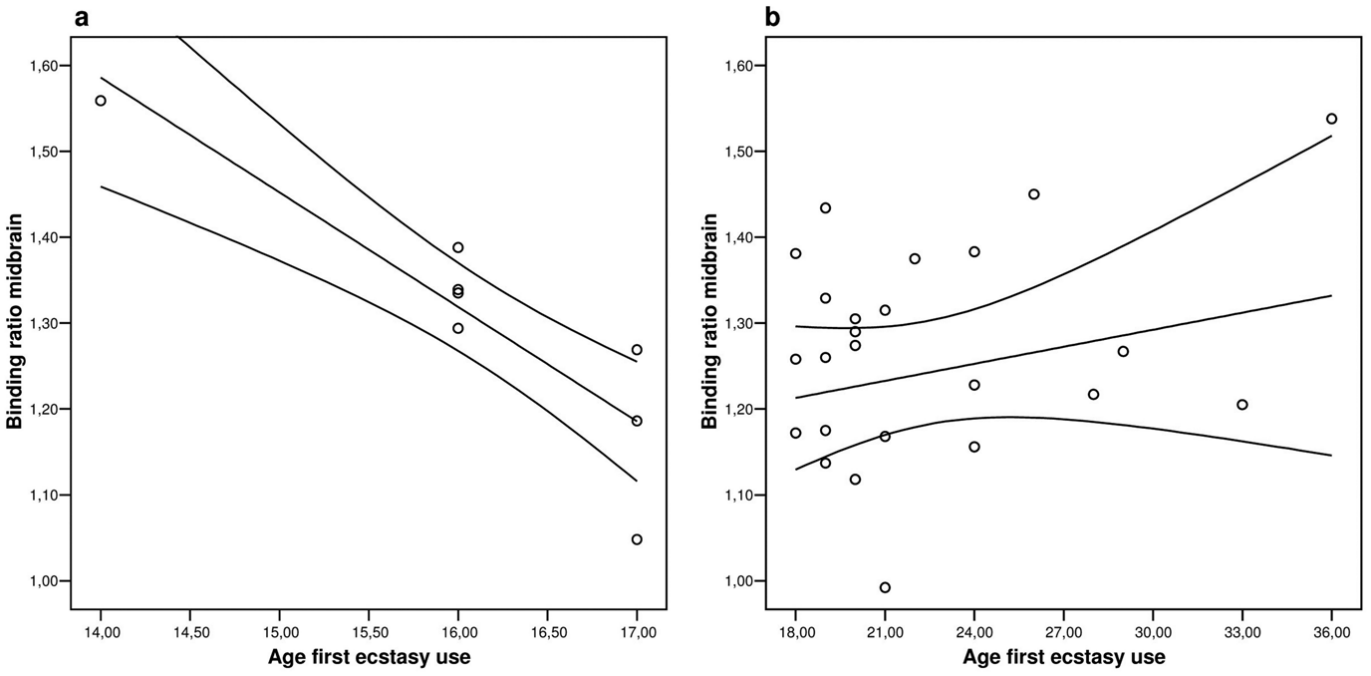
Early age of ecstasy use predicts higher SERT density in the developing brain. Midbrain SERT binding ratio plotted against age-at-first ecstasy exposure during *a*) brain development (first ecstasy use before 18 years; r^2^ = 0.789, r = −0.888, p = 0.003) and in *b)* in the mature brain (first ecstasy use 18 years or older; r^2^ = 0.032, r = 0.179, p = 0.392). 95% confidence limits are included with the regression line.

Midbrain [^123^I]ß-CIT binding ratios also did not differ between the two groups (1.30±0.15 in the early exposure group vs. 1.24±0.17 in the late exposure group, p = NS), nor frontal cortex (0.77±0.06 in the early exposure group vs. 0.77±0.08 in the late exposure group, p = NS). Analysis of variance revealed only a significant effect of age-at-first ecstasy use on midbrain [123I]ß-CIT binding ratios (p<0.01), whereas other possible confounding variables had no significant effect (e.g. age-at-time of scanning p = 0.37, gender p = 0.65, duration of ecstasy use p = 0.63, lifetime total amount of tablets p = 0.24). As postulated, correlation coefficient and regression analysis revealed a significant and very strong inverse relationship between age-at-first ecstasy use and midbrain [^123^I]ß-CIT binding ratios. However, this effect was observed in the early-exposed group (Fig. 1*a*) but not the late-exposed group (Fig. 1*b*), opposite to our hypothesis. Age-at-first use accounted for a notable 79% of the variability in [^123^I]ß-CIT binding ratios in the early-exposed group (Fig. 1*a*), whereas this was only 0.3% in the late-exposed group (Fig. 1*b*). Exclusion of potential outliers did not affect the results. More specifically, the exclusion of the 14 year-old age-of-onset subject did not significantly change the slope of the regression line. As expected, no such relationship between age-at-first ecstasy use and [^123^I]ß-CIT binding ratios was observed in the frontal cortex.

### SERT Binding in Rats

Specific SERT binding ratios are given per group and per region in Table 2.

**Table 2:** Specific [^123^I]β-CIT SERT binding ratios in saline and MDMA-treated rats.

	Control	MDMA	Delta	p-value
**Adolescent-treated**				
*Cortical regions*				*<0.01*
Prefontal cortex	3.55 (0.52)	2.29 (0.24)	−35%	<0.01
Temporal cortex	2.30 (0.26)	1.67 (0.18)	−27%	<0.01
Occipital cortex	2.57 (0.26)	1.60 (0.16)	−38%	<0.01
*Subcortical regions*				*<0.01*
Amygdala	5.87 (0.93)	3.64 (0.67)	−38%	<0.01
Hippocampus	3.44 (0.38)	2.13 (0.27)	−38%	<0.01
Hypothalamus	6.99 (1.03)	5.60 (1.07)	−20%	NS
Midbrain (sup. coll.)	7.06 (1.31)	2.16 (0.45)	−69%	<0.01
Striatum	17.40 (4.54)	20.93 (5.58)	+17%	NS
**Adult-treated**				
*Cortical regions*				*<0.01*
Prefontal cortex	4.47 (0.44)	2.27 (0.36)	−49%	<0.01
Temporal cortex	2.67 (0.40)	1.68 (0.22)	−37%	<0.01
Occipital cortex	3.03 (0.69)	1.75 (0.20)	−42%	<0.01
*Subcortical regions*				*<0.01*
Amygdala	6.50 (0.85)	3.58 (0.70)	−45%	<0.01
Hippocampus	3.76 (0.46)	2.20 (0.33)	−41%	<0.01
Hypothalamus	6.66 (0.79)	4.36 (1.08)	−35%	<0.01
Midbrain (sup. coll.)	8.08 (1.40)	2.0 (0.62)	−75%	<0.01
Striatum	16.61 (3.10)	22.56 (7.73)	+26%	NS

#### Effect of age on SERT binding

As expected, [^123^I]ß-CIT binding ratios in the prefrontal cortex of control adult rats were higher when compared to adolescent control rats (4.47 versus 3.55, respectively, +21%; p<0.01). In other brain regions, such as the raphe nuclei contained in the midbrain, no significant differences in [^123^I]ß-CIT binding ratios between adolescent and adult rats were observed, including the striatum (see also Table 2).

#### Effect of treatment

ANOVA analysis showed a significant main effect of treatment on cortical brain regions (p<0.01) and subcortical regions (p<0.01). Post hoc analysis revealed significant reductions in [^123^I]ß-CIT binding ratios in all 5-HT-rich brain regions studied in MDMA treated rats, except for the hypothalamus in the adolescent rats (−20%, p = NS). The reductions were less pronounced in adolescent rats when compared to adult rats: ranging from −20% to −69% in adolescent rats (on average −37%), and −35% to −75% in adult rats (on average −46%). In the striatum, [^123^I]ß-CIT binding ratios increased statistically non-significantly by 17% in adolescent and 26% in adult rats.

#### Age-BY-treatment interaction effects

A significant interaction effect of age-BY-treatment was observed in cortical brain regions (p<0.05), but not in subcortical brain regions or the striatum. As expected, post hoc analysis revealed that the effects of MDMA treatment on [^123^I]ß-CIT binding ratios were significantly different between adolescent and adult rats in the prefrontal cortex (p<0.01). In adolescent rats the degree of SERT loss in this brain region was less extensive when compared to adult rats following MDMA treatment (−35% versus −49%).

## Discussion

Age-at-first ecstasy use predicted midbrain SERT binding in ecstasy users that started to use during adolescence but not in users that started during adulthood. In adolescent rats, MDMA administration resulted in a less pronounced loss of SERT binding in the frontal cortex. The differences between the effects of MDMA on the developing and matured brain most likely occur because of the differential maturational stage of the 5-HT projection fields at the time of first exposure in combination with 5-HT’s neurotrophic effects on the connective organisation of the developing brain.

Our data suggest that in rats a normal developmental increase in SERT density renders the *frontal cortex* sensitive to MDMA’s age-related effects (a statistically significant increase of +21% in SERT density between control adolescent and adult rats was observed in this brain region). This observation is in line with other studies that report a steady increase in SERT density from weaning until old age in this brain region [22]. The frontal cortex is an ontogenetically later maturing brain region, and age-dependent increases in the densities of monoaminergic afferents may even continue to proceed until late adulthood in rat. The midbrain, on the other hand matures very early in rat, already during weaning [22], and indeed no age-BY-treatment effects were observed in this (or other) brains regions studied. It seems therefore likely that, in accordance with our hypothesis, the effects of MDMA in rats are (at least in part) determined by normal age-associated SERT densities. Other studies reported similar smaller reductions in SERT densities after early MDMA treatment (PND40: −21%) when compared to adult (PND70: −62%) treatment [12], [13] in the frontal cortex. In the present study, we observed a smaller difference between early and late MDMA treatment compared to Broening et al. [12] study, probably due to the younger age used in the present study (PND27 versus PND40). In the human study, we failed to find such an effect of age in the frontal cortex, as hypothesized. Besides a lack of age-associated changes in SERT densities in the human frontal cortex as pointed out earlier, another explanation for the species difference is that our sample of rat frontal cortex included the barrel field of primary somatosensory cortex (S-1). In this area of cortex, which receives input from the rat’s vibrissae, the thalamocortical afferents transiently express SERT during postnatal development as late as PND30 [42]. If these SERT-expressing but non-serotonergic fibers are not damaged by MDMA, then their presence at adolescence (PND27) but not in adulthood (PND67) might contribute to the less pronounced treatment effect at the earlier time point. Barrels are found in some species of rodents and species of at least two other orders [43], but not in humans.

In line with our hypothesis, we observed no age-related effects of MDMA in rat *midbrain,* due to early maturation of this brain region in rat. However, in the human midbrain, we observed a significant effect of age-at-first ecstasy use on SERT densities, in early-exposed individuals, and not late-exposed individuals as hypothesized. We expected less pronounced effects of MDMA in the late-exposed individuals due to documented age-associated reductions in SERT only after the age of 18 [27], [28]. The most likely explanation is that MDMA treatment does not cause loss of SERT in the midbrain due to 5-HT’s neurotrophic effects during development. Besides, the midbrain contains also the raphe nuclei. We previously observed that SERT density in the raphe nuclei, but not midbrain, is unaffected by MDMA in adult rat brain [44], an observation consistent with previous autoradiography [45], [46] and immunohistochemical [47], [48] studies. Thus, whereas the effect of age-at-first exposure of MDMA on 5-HT projection regions indeed may be primarily determined by age-associated SERT densities, in the midbrain its effects are also determined by 5-HT’s neurotrophic effects, due to proximity of the raphe nuclei.

5-HT’s neurotrophic effects on the midbrain are age and species related, and strongest in early exposed individuals. It is well known that 5-HT has neurotrophic effects before the maturation of its neurotransmitter function [49]–[51]. 5-HT’s neurotrophic effects on the midbrain containing the raphe nuclei are particularly strong. In line with this, MDMA, which is a potent 5-HT releaser, has been shown to increase 5-HT neuronal maturation in cell cultures of fetal raphe nuclei [9]. In addition, other drugs that induce high levels of 5-HT during brain development may also cause enhanced outgrowth of the 5-HT system [4]–[7]. In fact, ascending 5-HT projections of 5-HT neurons in the raphe nuclei have an enormous degree of structural plasticity, even in the adult brain: adult non-human primates with documented denervated proximal 5-HT projection fields such as the thalamus and amygdala, completely reinnervated or hyperinnervated 7 years post-MDMA treatment [47]. It has been suggested that release of a growth factor from astroglial cells in response to stimulation of postsynaptic 5-HT receptors causes this increased outgrowth of the 5-HT system [9], [52].

Several potential limitations of the current study should be mentioned. First, given the affinity of [^123^I]β-CIT for both 5-HT and dopamine transporters, our findings cannot be definitively ascribed to changes in the densities of SERT: the midbrain, thalamus, and cortex also contain dopamine transporters. However, displacement studies in animals have shown that binding of β-CIT is predominantly associated with SERT in these brain regions [35], [53]. As reported previously [11], we did not observe differences in striatal [^123^I]β-CIT binding ratios (obtained 24 h after injection of the radiotracer and reflecting binding to dopamine transporters) between heavy MDMA users and controls, also not in the present rat study. We therefore conclude that the findings probably reflect differences in densities of SERT and not dopamine transporters [11]. Second, as with all retrospective studies, there is a possibility that preexisting differences between the early and late-exposed group underlie differences in SERT densities–e.g., people with high SERT densities might be predisposed to start using ecstasy at a younger age. Because our sample size was small, the current findings need to be replicated in a larger cohort along with functional 5-HT outcome measures.

Our findings confirm previous animal findings that MDMA affects the developing brain differently and extend these observations for the first time to humans. These age-related effects most likely reflect the maturational stage of the 5-HT projection fields at age-at-first exposure and enhanced outgrowth of the 5-HT system due to 5-HT’s neurotrophic effects. At what age the 5-HT system becomes fully sensitive the MDMA’s neurotoxic effects is dependent on the developmental status of SERT and the maturation of 5-HT’s neurotransmitter function. These findings support the notion that during brain development the degree of structural plasticity of ascending 5-HT projections is higher than at later stages. To what extent these findings can be extrapolated to other drugs of abuse and medicines that have their primary action on the 5-HT system, is difficult to predict. Ultimately our data illustrate the need for more knowledge on the effects of pharmacotherapies that increase 5-HT levels during brain development, such as SSRIs for the treatment of childhood depression and anxiety disorders.
